# When Self-Reliance Is Not Safe: Associations between Reduced Help-Seeking and Subsequent Mental Health Symptoms in Suicidal Adolescents

**DOI:** 10.3390/ijerph120403741

**Published:** 2015-04-01

**Authors:** Christa D. Labouliere, Marjorie Kleinman, Madelyn S. Gould

**Affiliations:** Division of Child and Adolescent Psychiatry, The New York State Psychiatric Institute, Columbia University Medical Center, 1051 Riverside Drive, New York, NY 10032, USA; E-Mails: labouli@nyspi.columbia.edu (C.D.L.); kleinmam@nyspi.columbia.edu (M.K.)

**Keywords:** help-seeking, self-reliance, adolescents, suicide, depression

## Abstract

The majority of suicidal adolescents have no contact with mental health services, and reduced help-seeking in this population further lessens the likelihood of accessing treatment. A commonly-reported reason for not seeking help is youths’ perception that they should solve problems on their own. In this study, we explore associations between extreme self-reliance behavior (*i.e.*, solving problems on your own all of the time), help-seeking behavior, and mental health symptoms in a community sample of adolescents. Approximately 2150 adolescents, across six schools, participated in a school-based suicide prevention screening program, and a subset of at-risk youth completed a follow-up interview two years later. Extreme self-reliance was associated with reduced help-seeking, clinically-significant depressive symptoms, and serious suicidal ideation at the baseline screening. Furthermore, in a subset of youth identified as at-risk at the baseline screening, extreme self-reliance predicted level of suicidal ideation and depressive symptoms two years later even after controlling for baseline symptoms. Given these findings, attitudes that reinforce extreme self-reliance behavior may be an important target for youth suicide prevention programs. Reducing extreme self-reliance in youth with suicidality may increase their likelihood of appropriate help-seeking and concomitant reductions in symptoms.

## 1. Introduction

Suicide is the third leading cause of death for adolescents, accounting for more deaths each year than all natural causes combined [1]. Nationwide each year, 13.8% of adolescents experience suicidal ideation and 5%–10% make a suicide attempt [1]. The risk of death by suicide is further elevated for those not receiving adequate treatment [2], which is alarming considering most suicidal persons have no contact with the mental health system [3]. Suicidal adolescents and young adults tend to seek help less frequently than older adults for their mental health problems, both from professionals and from more informal sources [4,5]. Although adolescent suicidality has serious risk for mortality and morbidity, mental health service utilization for suicidal adolescents is markedly low [5,6,7,8], and those with the most severe symptoms are often the least likely to seek help [4,9]. In response, school-based programs have been developed that seek to identify youth who are at-risk, while also trying to change attitudes and beliefs that may be barriers to help-seeking [10,11,12]. 

Ultimately, attitudes about help-seeking remain a primary predictor of formal mental health service utilization [13], and adolescents with negative attitudes about help-seeking are frequently reticent to disclose their symptoms, especially to adults. The fear that confidantes may betray their confidentiality; deep concerns that their peers, teachers, or families may learn of their symptoms, judge them negatively (*i.e.*, stigma), or treat them differently or discriminatorily; and hopelessness that treatment could be effective have all been identified as important barriers to help-seeking in suicidal adolescents [14], although resource access and mental health literacy likely also play an important role [9,14,15,16,17]. However, less attention in the literature has been paid to self-reliance, or the perception that a person should be able to solve their problems on their own. 

Suicidal adolescents frequently express that needing help from others is a failure or weakness, and that they should be able to address their problems without external assistance [17,18,19,20]. Extreme levels of self-reliance have been identified as a barrier to help-seeking more generally in other mental health, educational, and medical environments [21,22,23,24,25], but few studies have directly examined extreme self reliance in regard to adolescent suicidality. Given the known propensity for cognitive rigidity frequently seen in suicidal adolescents [26], these extreme views on the value of self-reliance may form a sort of “self-stigma”, wherein adolescents’ negative attitudes about help-seeking and extreme self-reliance behavior prevent them from disclosing their symptoms or engaging in professional treatment even in the face of dangerous mental health symptoms [27,28,29]. 

Despite the importance to help-seeking decisions of the value placed on self-reliance, the prevalence of extreme levels of self-reliance and its relationship to mental health symptoms in adolescence is not well understood. As such, in this study, we explore associations between extreme self-reliance, help-seeking behavior, and mental health symptoms in a community sample of 2342 adolescents participating in a school-based suicide screening program. Adolescents identified as at heightened risk for suicidality at the baseline assessment were followed for two years so that the prospective predictive value for extreme self-reliance could be determined. We hypothesized that: (1) youth endorsing extreme levels of self-reliance will have more symptoms of depression and suicidality than youth with less extreme help-seeking behavior; and (2) youths’ endorsement of extreme level of self-reliance behavior at the baseline assessment will predict continued symptoms of depression and suicidality over a two-year follow-up, controlling for baseline symptomatology. The findings from this study may enhance our ability to develop more effective suicide prevention programs, directly targeting extreme self-reliance as a barrier to help-seeking and assisting suicidal youth to overcome their “self-stigma” and accept/seek help more readily.

## 2. Methods

### 2.1. Participants 

*Baseline.* Data for this study were collected as part of a school-based suicide prevention screening program offered to ninth- through twelfth-graders at six schools (five co-educational public and one all-boys parochial) in three suburban counties of New York. These schools were part of a study examining whether assessment of suicidality produced iatrogenic effects [29]. All participants’ parents were informed via mail in advance of the screening and were given the opportunity to opt-out of permitting their children to participate (*i.e.*, passive consent). Immediately before screening, all adolescent participants provided written informed assent (see [29] for more information). The study was conducted in accordance with the Declaration of Helsinki, and the protocol was approved by school administrators and the Institutional Review Board of the New York State Psychiatric Institute at Columbia University Medical Center (Protocol #4264R).

Data collection utilized a group-randomized design, wherein individual classrooms within a school were assigned to either an experimental or control group. On Day 1, students in experimental classrooms received a first screening survey that included assessment of suicidal ideation and behavior, whereas students in control classrooms did not answer any questions about suicidality on their first screening survey. Students in both experimental and control classrooms received a second screening survey on a subsequent day (Day 2) that included questions assessing suicidality. Of the 2342 participants, 2145 had data from both Day 1 (when help-seeking behavior was assessed) and Day 2 (when depression and suicidality were assessed for all participants). No significant differences were noted between those who did and did not complete both surveys on age, race, or ethnicity, although those who did not complete both surveys were somewhat older (15.5 years *vs.* 14.8 years; *t*_2340_ = 7.78, *p* < 0.001). The final baseline sample of 2145 participants was 58.1% male and had a mean age of 14.8 (*SD* = 1.18) years at baseline; 51.6% of participants were freshmen, 25.5% sophomores, 14.4% juniors, and 8.6% seniors. Approximately 20% identified as ethnic or racial minorities (4.6% African American, 8% Hispanic, 3.2% Asian, and 3.1% another group), consistent with the demographic composition where data was collected. 

*Follow-Up.* Three hundred seventeen youth were identified as at-risk for suicide. A youth was designated “at-risk” during the baseline screen if on either day he or she: (a) reported serious suicidal ideation, as operationalized by a score past the critical cut-off value of 31 on the Suicide Ideation Questionnaire, Junior Version (SIQ-JR) [30]; (b) endorsed any of six SIQ-JR “critical items” at clinically significant levels (*i.e.*, selected “a couple times a week” or “almost everyday” for the questions “I thought about killing myself”, “I thought about how I would kill myself”, “I thought about what to write in a suicide note”, “I thought about writing a will”, or “I thought about telling people I had a plan to kill myself”); (c) endorsed the statements “I would like to kill myself” or “I would kill myself if I had the chance” on the Beck Depression Inventory-IA (BDI-IA) [31]; (d) reported a past suicide attempt, regardless of timing, lethality, or need for medical attention; or (e) demonstrated clinically-significant depressive symptoms, as operationalized by a score past the cut-off value of 16 on the BDI-IA. These risk criteria were based on those identified in psychological autopsy studies of youth who died by suicide [32,33], and have been used in other published studies of youth at risk for suicide [8].

At-risk youth were subsequently followed for approximately two years after the initial screening, with the average number of days between the initial survey and follow-up being 750 (range 519–1207 days). Approximately 57% of the youth originally identified as at-risk for suicide participated in the two-year follow-up (*N* = 180, with *N* = 179 having complete data). There were no significant differences between participants and non-participants at follow-up in terms of age, race, ethnicity, grade, functional impairment, mental health symptoms, help-seeking, or self-reliance behavior at baseline, although follow-up participants were more likely to be female (**χ**^2^ = 7.35, *p* < 0.01). The final follow-up sample of 179 participants was 66.1% female, had a mean age of 15.0 (*SD* = 1.20) years at baseline, and identified as 19.4% racial or ethnic minority (3.3% African American, 11.1% Hispanic, 1.7% Asian, and 3.3% another group).

### 2.2. Measures

*Demographics*. Age, grade, gender, and racial/ethnic background were assessed.

*Suicidal Ideation Questionnaire-Junior Version (SIQ-Jr.).* The SIQ-Jr. [30] is a 15-item questionnaire that assesses the frequency of specific suicidal thoughts during the preceding four weeks. The measure uses a 7-point Likert scale, ranging from 0 (“never had this thought”) to 6 (“this thought was in my mind almost every day”), to assess a wide range of thoughts related to death, dying, passive and active suicidal ideation, and suicidal intent. The SIQ-Jr. demonstrates excellent psychometric properties, with internal consistencies ranging from 0.91–0.96, test-retest associations between 0.87–0.93, and strong demonstrations of validity in both community and clinical adolescent populations [30,34,35,36]. Continuous measures of *suicidal ideation* utilized SIQ-Jr. total scores, whereas categorical measures of *clinically-significant suicidal ideation* were derived from clinical cut-off scores and critical items (see “Defining Variables” subsection of the “Analytic Strategy” section for more details). 

*Suicide Attempt History.* Seven questions assessing lifetime and recent suicide attempts, injuries sustained, medical care sought, and psychiatric hospitalization were adapted from the depression module of the Diagnostic Interview Schedule for Children-IV [37] and the Columbia Suicide Screen [38]. These items have demonstrated good validity in other studies [8,29].

*Beck Depression Inventory (BDI-IA).* The BDI-AI [31,39] is a 21-item measure assessing cognitive, behavioral, affective, and somatic symptoms of depression. The measure uses a 4-point scale, ranging from 0 (symptom is not present) to 3 (symptom is severe). The BDI has a long history of strong psychometrics, with internal consistencies ranging from 0.8–0.9, good test-retest associations (0.7), and excellent specificity and sensitivity in research with adolescents [40,41,42]. Continuous measures of *depressive symptoms* utilized BDI-IA total scores, whereas categorical measures of *clinically-significant depression* were derived from clinical cut-off scores (see “Defining Variables” subsection of the “Analytic Strategy” section for more details). 

*Help-Seeking Utilization Questionnaire (HUQ).* The HUQ [8,43] is a 13-item adaptation of Offer’s Mental Health Utilization Questionnaire [44], assessing the frequency with which a youth sought help from formal and informal sources in the month prior to the baseline assessment. The HUQ has shown good validity in previous studies with adolescents [8,43]. The measure uses a 5-point scale, ranging from 0 (“none of the time”) to 4 (“all of the time”), where each item represents a different formal or informal help-seeking source (parents, siblings, friends, teachers, coaches, crisis hotline, internet, alcohol or drug rehab program, medical professional, mental health professional, school counselor, or clergy). *Help-seeking behavior* variables represent the frequency of seeking help from each source in the past month (0–4), with each source reported by a separate, single item on the HUQ. A single item on the HUQ also assesses whether youth “solved problems on their own” in the past month. Youth were designated as endorsing *extreme self-reliance* if they endorsed the most extreme value on that item (*i.e.*, reported that they solved their problems on their own “all the time”). 

*Current treatment.* Whether a youth was currently receiving treatment from a mental health professional was assessed with a single dichotomous item (Yes/No), as has previously been reported in prior studies [29].

### 2.3. Procedure

Students were recruited for the suicide prevention screening program with an “opt-out” procedure for parents and written assent from youth. Two mailings with information about the program and questionnaire content were sent to families six and four weeks before screening administration. These mailings contained a response form and stamped envelope so that parents/guardians were provided an opportunity to refuse their child's participation. Youth provided written assent immediately before administration of screening measures. At baseline, participants completed measures of help-seeking behavior, depressive symptoms, and suicidal behavior and ideation in class over the course of two days as part of a school-based suicide prevention screening program, as previously described [29].

Youth identified as at-risk for suicide at baseline were evaluated by a project child/adolescent psychologist, psychiatrist, or social worker to assess imminent suicide risk and the need for further evaluation and treatment. Project social workers contacted the parents/guardians of the at-risk youth, explained screening results, and discussed recommendations for further evaluation and treatment with local mental health professionals. All families were offered provider lists and specific referrals, taking into consideration the youth's specific symptoms and insurance status; however, some families did not accept these lists as they already were involved with mental health treatment or did not feel that treatment was necessary. Regardless of whether they accepted referral information, all families were given the project social worker's contact information in case they desired assistance in the future. If families were willing, parent/guardian follow-through with contacting mental health professionals for their child was tracked until the first appointment had been attended or it became clear after an extended period of time that the family did not intend to pursue services. 

Youth who were identified as at-risk for suicide at baseline were followed for approximately two years. After approximately two years, families were re-contacted by telephone and asked if they were willing to participate in a follow-up interview. Follow-up interviews were conducted with youth participants via telephone after consent and assent were obtained. All recruitment and consent procedures were approved by school administrators and the Institutional Review Board of the New York State Psychiatric Institute. 

### 2.4. Analytic Strategy

*Defining variables.* Youth were designated as endorsing *extreme self-reliance* if they reported that they solved their problems on their own “all the time” (most extreme value) on the HUQ. *Help-seeking behavior* variables represent the frequency of seeking help from each source in the past month, with each source reported by a separate item. Whether youths were *currently in treatment* with a mental health professional was derived from a single dichotomous item assessing current treatment utilization. Continuous measures of *depressive symptoms* and *suicidal ideation* utilized total scores on the BDI-A and SIQ-Jr., respectively. Categorical measures of *clinically-significant depression* and *clinically-significant suicidal ideation* were derived from clinical cut-offs and critical items on the BDI-A and SIQ-Jr. Youth were included in the Clinically-Significant Depression group if they obtained a score past the clinical cut-off of 16 on the BDI-IA. Youth were included in the Clinically-Significant Suicidal Ideation group if they reported a history of suicide attempts, scored past the clinical cut-off of 31 on the SIQ-Jr., or endorsed a critical item on the SIQ-Jr. or the suicide item on the BDI-IA at clinically significant levels (*i.e.*, 5–6 on the SIQ-Jr. and 2–3 on the BDI-IA). 

*Analyses.* The primary sampling unit was school and the secondary sampling unit was student within school. Thus, we first examined the extent of within-school clustering to determine whether this clustering variable warranted inclusion in our analyses. The sample clusters (school) had little impact on the outcomes (HUQ, SIQ-Jr., BDI), as indicated by the intraclass coefficients, which were all close to zero. Therefore, the use of mixed-effects linear models to account for the clustering variable of school was unnecessary. All analyses utilized a modified Bonferroni adjustment for multiple testing, covaried for school and gender, and had adequate statistical power to detect small effects. Statistical analyses were conducted using SAS version 9.4 (SAS Institute, Inc.; Cary, NC, USA) and SPSS version 20 (IBM, Inc.; Chicago, IL, USA).

Multivariable linear regression models were used to determine differences in mental health symptoms and help-seeking behavior between youth who did and did not endorse extreme self-reliance at the baseline assessment. Random effects of school and fixed effects of gender were included in all models, and eta-squared was calculated as a measure of effect size. Eta-squared (η^2^) is a measure of effect size in ANOVA or mixed-model general linear modeling that is analogous to *R*^2^ in linear regression. Eta-squared represents the percentage of variance accounted for by a given variable in a model, ranges from 0-1, and can be interpreted in a similar fashion to r^2^, where 0.01 denotes a small effect, .06 a moderate effect, and .14 a large effect in one-way modeling [45,46]. Continuous measures of depression (*i.e.*, BDI-IA total scores), suicidal ideation (*i.e.*, SIQ-Jr. total scores), and help-seeking (*i.e.*, frequency of seeking help from each source, 0–4 per item) were used as outcomes in the linear regression models, with separate models conducted for each mental health symptom and help-seeking source. Categorical analyses were also conducted to supplement continuous analyses, with continuous measures used to denote differing levels of symptomatology across a spectrum of severity and categorical measures used to denote symptom severity past a clinically-significant threshold. To evaluate the differences in endorsement of extreme self-reliance between youth with and without clinically-significant depression or clinically-significant suicidal ideation, chi-square tests with Mantel-Haenszel odds ratios were used. 

Lastly, in the subsample of youth identified as at heightened risk for suicidality at baseline, follow-up multivariable linear regression models used endorsement of extreme self-reliance at baseline as a predictor of depression (BDI-IA scores) and suicidal symptoms (SIQ-Jr. scores) at two-year follow-up, controlling for baseline levels of symptomatology. 

## 3. Results

In preliminary analyses, gender differences were noted in mental health symptoms and help-seeking behaviors; as expected based on the literature, female participants had higher rates of mental health symptoms and help-seeking behaviors (see Table 1). As such, gender was included as a covariate on all subsequent analyses. Of the 2140 youth participants, 16.6% endorsed extreme levels of self-reliance behavior in the past month at the baseline assessment; no significant differences in rates of endorsement existed across gender, race, ethnicity, or school. 

Youth endorsing extreme levels of self-reliance had significantly higher depression scores than youths with less extreme help-seeking behavior (*F*_3,2135_ = 100.27, *p* < 0.0001, η^2^ = 0.05) and were three times more likely to meet criteria for clinically-significant levels of depression (22% *vs.* 9%; χ^2^ = 50.11, *p* < 0.001, OR = 2.84; 95% CI = 1.82–3.76). Furthermore, youth endorsing extreme self-reliance also had significantly higher suicidal ideation scores (*F*_3,2135_ = 73.00, *p* < 0.0001, η_p_^2^ = 0.03), and their odds of meeting criteria for clinically-significant levels of suicidal ideation were nearly 2.5 times greater than for youth not endorsing extreme self-reliance (13.6% *vs.* 5.7%; χ^2^ = 28.39, *p* < 0.001, OR = 2.61; 95% CI = 2.11–3.84; see Table 1). 

As expected, youth endorsing extreme levels of self-reliance had significantly lower odds of reaching out to traditional informal sources of help, such as parents (*F*_1,2131_ = 22.33, *p* < 0.001, η^2^ = 0.02) or friends (*F*_1,2131_ = 6.82, *p* < 0.01, η^2^ = 0.02), but were more willing to seek support from anonymous electronic sources, such as internet forums, support groups, or chat rooms (*F*_1,2131_ = 7.41, *p* < 0.01, η^2^ = 0.01; see Table 1). There were no other differences in informal sources of help (*i.e.*, siblings, teachers, coaches, crisis hotline, alcohol or drug rehab program, medical professional, school counselor, or clergy) between those with extreme self-reliance and those with more moderate help-seeking behavior, likely due to equivalent levels of low usage across both groups (range: 1%–16% endorsement).

**Table 1:** Baseline mental health symptoms and help-seeking behavior of adolescents who did and did not endorse extreme self-reliance.

Variables	Total (*N* = 2140)	Gender			Extreme Self-Reliance		
		Male (*n* = 1243)	Female (*n* = 896)	*p*	Yes (*n* = 356)	No (*n* = 1783)	*p*
**Mental Health Symptoms**							
Depression symptoms *(continuous)*	6.87 (6.96)	5.79 (5.88)	8.47 (8.00)	0.001	10.05 (8.70)	6.28 (6.39)	0.0001
Clinically-significant depression *(categorical)*	11%	7.1%	16.8%	0.001	22.0%	9.0%	0.001
Suicidal ideation *(continuous)*	6.42 (10.56)	5.07 (9.08)	8.38 (12.08)	0.001	10.60 (14.65)	5.64 (9.34)	0.0001
Clinically-significant suicidal ideation *(categorical)*	7%	5%	10%	0.001	13.6%	5.7%	0.001
**Current Treatment**	6.5%	5.2%	8.3%	0.01	9.3%	5.9%	0.05
**Help-Seeking Behavior**							
Parent	63.5% 1.26 (1.23)	58.9% 1.14 (1.20)	69.9% 1.41 (1.25)	0.001	48.0% 0.97 (1.24)	66.6% 1.31 (1.22)	0.001
Friend	74.9% 1.78 (1.39)	62.8% 1.26 (1.25)	92.0% 2.51 (1.24)	0.001	65.9% 1.61 (1.48)	76.9% 1.82 (1.37)	0.01
Internet	4.4% 0.07 (0.37)	4.3% 0.07 (0.39)	4.6% 0.07 (0.35)	0.81	7.1% 0.12 (0.50)	3.9% 0.06 (0.34)	0.01
Mental health professional	6.9% 0.14 (0.57)	4.8% 0.09 (0.45)	9.7% 0.20 (0.70)	0.001	9.6% 0.24 (0.78)	6.4% 0.12 (0.52)	0.001

**Note:** Extreme self-reliance was defined as answering the question “In the past four weeks when you’ve been feeling stressed, upset, or sad, how often did you solve problems on your own?” with the most extreme value, “All of the time”. Extreme self-reliance did not differ significantly by gender (χ^2^ = 2.38, *p* = 0.12). Continuous mental health symptoms were based on BDI-A and SIQ-Jr. scores and are reported as mean (SD). Categorical mental health symptoms (*i.e.*, at a clinically-significant level) are reported as %-endorsing. Help-seeking data are reported as %-endorsing using a source of support at a nonzero level in the past month (1st line) and mean for frequency of contact (SD) on a 5-point scale ranging from “Not at all” (0) to “all of the time” (4; 2nd line). Sources of help-seeking that did not significantly differ between groups (likely due to low levels of endorsement across both groups) are omitted. Higher scores indicate higher values of that construct. Random effects of school and fixed effects of gender were included in all models. One student did not identify as male or female and was not included in analyses of gender differences.

Interestingly, youth with extreme self-reliance had significantly greater odds of currently being in treatment (χ^2^ = 5.74, *p* < 0.05, OR = 1.64; 95% CI = 1.09–2.47) than their peers with less extreme help-seeking behavior, and therefore endorsed more frequent help-seeking from a mental health professional (*F*_3,2132_ = 13.77, *p* < 0.001; η^2^ = 0.01). However, when only youth currently receiving treatment were included in analyses, youth with extreme self-reliance reported seeking help from their mental health professional significantly less frequently compared to other youth in treatment (*M* = 1.32 *vs.* 1.76; *F*_3,138_ = 5.02, *p* < 0.05; η^2^ = 0.04), despite having similar levels of depressive symptoms (*F*_3,137_ = 2.62, *p* = 0.07) and suicidal ideation (*F*_3,138_ = 1.69, *p* = 0.20). Furthermore, when depression and suicidal ideation symptoms were entered into a hierarchical general linear model before extreme self-reliance, self-reliance no longer remained a significant predictor of current treatment (*B*(*SE*) = −0.009 (0.33), Wald = 0.001, *p* = 0.97) or frequency of help-seeking from a mental health professional (*β* = 0.02, *t* = 0.81, *p* = 0.42). Taken together, these results suggest that youth endorsing extreme self-reliance may have been more likely to be in treatment due to the severity of their symptoms rather than a choice based on their values.

Among the subsample of 179 youth identified as being at heightened risk for suicidality at baseline who also participated in a two-year follow-up, 29.1% endorsed extreme self-reliance at baseline. These youth had significantly higher depression scores at follow-up than youths with less extreme help-seeking behavior (*F*_4,179_ = 5.85, *p* < 0.05, η^2^ = 0.03), even when controlling for baseline levels of depression. Likewise, while controlling for baseline levels of suicidality, youth endorsing extreme self-reliance had significantly higher suicidal ideation scores at follow-up (*F*_4,177_ = 4.70, *p* < 0.05, η^2^ = 0.03; see Table 2). 

**Table 2:** Mental health symptoms at two-year follow-up of adolescents identified as at heightened risk for suicidality at baseline who did and did not endorse extreme self-reliance.

Follow-Up Mental Health Symptoms	Total (*N* = 179)	Gender			Extreme Self-Reliance		
		Male (*n* = 60)	Female (*n* = 119)	*p*	Yes (*n* = 52)	No (*n* = 127)	*p*
Depression symptoms	10.08 (7.28)	7.92 (5.14)	11.18 (7.95)	0.01	12.42 (8.94)	9.12 (6.49)	0.05
Suicidal ideation	9.19 (9.71)	7.64 (9.95)	9.98 (9.53)	0.13	11.67 (12.45)	8.16 (8.28)	0.05

**Note:** Extreme self-reliance was defined as answering the question “In the past four weeks when you’ve been feeling stressed, upset, or sad, how often did you solve problems on your own?” with the most extreme value, “All of the time”. Extreme self-reliance did not differ significantly by gender (χ^2^ = 0.30, *p* = 0.58). Mental health symptoms were based on BDI-A and SIQ-Jr. scores and are reported as mean (SD). Higher scores indicate higher values of that construct. Random effects of school and fixed effects of gender were included in all models.

## 4. Discussion 

In this study, we explored the associations between extreme self-reliance behavior (*i.e.*, solving problems entirely on your own all the time), and mental health symptoms in a large sample of high-school students. Extreme self-reliance was endorsed by 16.6% of the total sample of adolescents, but by 29.1% of youth identified as being at heightened risk for suicide. Extreme self-reliance was associated with clinically-significant levels of depression and suicidal ideation, both at the baseline assessment and at a two-year follow-up assessment with youth identified as being at heightened risk for suicide.

Consistent with previous studies of adolescents, the majority of our sample had predominantly healthy attitudes about help-seeking [10,27]. Approximately 64% of youth reported turning to parents for support at least some of the time in the last month, and nearly 75% reported turning to a friend, rates similar to those found in other studies [22,27,44]. Also in concert with previous studies were gender differences in mental health symptoms and help-seeking behavior, with female participants endorsing higher rates than male participants [22,27,28,44]. While these gender differences were to be expected based on the literature, extreme self-reliance behavior showed no differential rate of endorsement across gender. While mental health symptoms and help-seeking behavior may be influenced by a number of gender-specific social, cultural, and biological factors in adolescence [13,28], it is possible that messages overvaluing self-sufficiency and individualism that contribute to extreme self-reliance may not vary by gender as strongly as expected. While such messages as traditionally viewed as being more relevant to masculine gender roles [47], it is possible that the more equalitarian perspective regarding women’s self-sufficiency and independence over the past several decades has made these messages increasingly salient to female adolescents, resulting in similar levels of male and female adolescents developing maladaptive, extreme views on the acceptability of help-seeking behavior.

The 17% of adolescents who endorsed extreme self-reliance at baseline reported reduced help-seeking behavior from informal sources traditionally favored by adolescents, such as friends or parents. This reduced help-seeking behavior was in stark contrast to their increased need, as youth who endorsed extreme self-reliance experienced significantly elevated symptoms of depression and suicidal ideation, and were 2–3 times more likely to experience depression and suicidal ideation at a clinically-significant level. 

It is somewhat disconcerting that those youth most likely to be in need of emotional support—youth with serious mental health symptoms—were also far more likely to rely only on themselves for these serious problems. Depression and suicidality frequently result in cognitive biases associated with reduced adaptive problem-solving [26], making these youth the least equipped to utilize effective coping skills in the face of their emotional distress [27]. Adolescents endorsing extreme self-reliance were less likely to seek assistance from parents or friends, and more likely to seek information from anonymous sources such as the Internet. While help-seeking from the Internet is ostensibly better than no help-seeking at all (as it can provide psychoeducation and some degree of emotional support), other studies have suggested that the majority of adolescents who turn to the Internet for support report it to be unhelpful [43]. Furthermore, the quality of information available on the Internet may not be as consistent or accurate as that provided by a knowledgeable adult such as a counselor or mental health professional [48]. Even worse, vulnerable youth may risk exposure to bullying or victimization at the hands of unscrupulous “Internet trolls”, persons who intentionally upset others or sow discord in online forums. Perhaps most importantly, adolescents who disclose suicidal thoughts or behaviors online may not have revealed enough identifying information for others to successfully intervene to ensure their safety in imminent situations given the relative anonymity of many Internet services. These downsides of Internet support are in contrast to disclosures made to friends or family, which are more likely to result in supportive interactions [21,49] or consultation with formal mental health treatment or emergency services if symptoms become too severe [5]. As reduced informal help-seeking lessens the likelihood of disclosure to those who may encourage or facilitate treatment [4], extreme self-reliance may result in lower chances of early intervention. 

Initially, it seemed somewhat counterintuitive that youth reporting extreme self-reliance were actually more likely to be in current treatment with a mental help professional compared to their peers with more moderate help-seeking behavior. However, further analyses with only youth currently in treatment revealed that youth with extreme self-reliance endorsed seeking help from their mental health professional significantly less frequently compared to other youth in treatment, despite having similar levels of mental health symptoms. Moreover, when level of mental health symptoms was taken into account, relationships between self-reliance and help-seeking from a mental health professional vanished. As such, it seems that the relationship between youth endorsing extreme self-reliance and mental health treatment is likely an artifact of severity of psychopathology rather than self-reliant youth electing to attend mental health treatment based on their attitudes and values. Youth are a unique population in regard to mental health treatment, as they traditionally do not choose to attend therapy of their own volition [50,51]. It is likely that many of these adolescents would not otherwise be treatment-seeking if they were not compelled to attend mental health services by concerned parents, family, or teachers. As such, our findings suggest that, while adolescents endorsing extreme self-reliance may be more likely to be brought to treatment due to their severe symptoms, these attitudes and behaviors may prevent them from fully engaging in the therapeutic process, resulting in reduced efficacy. 

While other studies have shown maladaptive attitudes about help-seeking in youth with suicidal symptoms [27], this study is the first to show the robust link between extreme self-reliance behavior and severity of mental health symptoms in suicidal adolescents. Furthermore, extreme self-reliance behavior predicted their level of suicidal ideation and depressive symptoms two years later, even after controlling for baseline symptoms, in the subset of youth identified as at-risk at baseline. While it cannot be said with certainty whether extreme self-reliance is a cause of the symptoms measured at baseline, these findings do suggest that extreme self-reliance plays an important role in maintaining these symptoms across adolescence, perhaps cutting adolescents off from social support and help-seeking efforts that could have ameliorating effects on their psychopathology. 

There were several limitations to this study. One limitation was the use of a convenience, rather than a random sample, of schools. As these schools were willing to participate in our screening program, there may have been differences in the attitudes and motivation of their administration or their organizational climate toward suicide prevention that could affect students’ perceptions of the appropriateness of help-seeking. Furthermore, while socioeconomically-diverse, these schools were predominantly suburban and composed of mostly Caucasian students, so results may not be generalizable to more diverse pupils or settings. Future studies should utilize more diverse samples, so that it can be determined if self-reliance varies based on racial or cultural expectations. Likewise, it is possible that attrition across measurement points may somehow have biased findings at follow-up, although this possibility seems less likely considering the consistency of the findings from baseline to follow-up and the similarity of participants and non-participants on mental health symptoms, help-seeking behavior and demographic variables (except gender) at baseline. A further limitation was that, due to the time constraints inherent to school-based mental health research, several of the variables were measured using single items or dichotomous (Yes/No) measures. Single-item and dichotomous item measurement reduces potential variability, thereby increasing the likelihood of error and reducing statistical power; as such, it is possible that smaller effects were unable to be detected due to these measurement issues. While the use of a large sample size at baseline partially mitigates this reduction in power, future studies should consider using more nuanced, multi-item scales in order to explore relationships in a more detailed manner, especially when smaller sample sizes are necessary (*i.e.*, exploring relationships in clinical subsets, across follow-up, *etc.*). Another limitation is the fact that other potentially-moderating variables of the relationship between self-reliance, help-seeking, and symptoms were not measured, such as perceptions of mental health stigma, barriers to help-seeking, previous experiences with help-seeking, hopelessness, expectancies about treatment efficacies, or identification with a culture or subculture with strong beliefs and expectations about individualism and self-sufficiency. Future studies should explore how these factors interact with extreme levels of self-reliance to predict help-seeking behavior in depressed and suicidal youth. However, despite these limitations, this study also had many strengths, most notably a large sample size, strong statistical techniques, and an important topic that was relatively unexplored in the adolescent suicide literature previously. These findings have important implications for therapeutic and public health interventions. 

## 5. Conclusions

Adolescents with extreme views on the value of self-reliance experience a sort of “self-stigma”, wherein their negative attitudes about help-seeking and extreme self-reliance behavior prevent them from disclosing their symptoms or engaging in professional treatment even in the face of dangerously elevated mental health symptoms. Given these findings, attitudes that reinforce extreme self-reliance behavior may be an important target for youth suicide prevention programs. Likewise, therapists working with depressed and suicidal youth should assess for the presence of these attitudes and behaviors so that they can be targeted in therapy and not interfere with alliance-building or diminish therapeutic effectiveness. Reducing extreme self-reliance behavior in youth with suicidality may increase their likelihood of appropriate help-seeking and concomitant reductions in symptoms.
